# Baseline predictors for subretinal fibrosis in neovascular age-related macular degeneration

**DOI:** 10.1038/s41598-021-03716-8

**Published:** 2022-01-07

**Authors:** Philipp K. Roberts, Markus Schranz, Alice Motschi, Sylvia Desissaire, Valentin Hacker, Michael Pircher, Stefan Sacu, Wolf Buehl, Christoph K. Hitzenberger, Ursula M. Schmidt-Erfurth

**Affiliations:** 1 Department of Ophthalmology and Optometry, Medical University of Vienna, Waehringer Guertel 18-20, 1090 Vienna, Austria; 2 Center for Medical Physics and Biomedical Engineering, Medical University of Vienna, Waehringer Guertel 18-20, 1090 Vienna, Austria

**Keywords:** Diagnostic markers, Prognostic markers, Biomarkers, Signs and symptoms

## Abstract

To find baseline predictors for subretinal fibrosis (SF) in neovascular age-related macular degeneration (nAMD). Forty-five eyes of 45 participants with treatment-naïve nAMD were consecutively enrolled and treated according to a standardized treat-and-extend protocol. Spectral-domain optical coherence tomography (OCT), color fundus photography and fluorescein angiography as well as novel imaging modalities polarization-sensitive OCT and OCT angiography (OCTA) were performed to detect SF after 1 year and find baseline predictors for SF development. Baseline OCTA scans were evaluated for quantitative features such as lesion area, vessel area, vessel junctions, vessel length, vessel endpoints and mean lacunarity. Additionally, the type of macular neovascularization, the presence of subretinal fluid, intraretinal fluid (IRF), subretinal hyperreflective material (SHRM), retinal hemorrhage as well as best-corrected visual acuity (BCVA) were evaluated. After 12 months 8 eyes (18%) developed SF. Eyes with SF had worse baseline BCVA (*p* = .001) and a higher prevalence of IRF (*p* = .014) and SHRM at baseline (*p* = .017). There was no significant difference in any of the evaluated quantitative OCTA parameters (*p* > .05) between eyes with and without SF. There were no quantitative baseline microvascular predictors for SF in our study. Low baseline BCVA, the presence of IRF and SHRM, however, are easily identifiable baseline parameters indicating increased risk.

## Introduction

Subretinal fibrosis (SF), together with macular atrophy, is the most important cause for poor visual outcome in neovascular age-related macular degeneration (nAMD)^1–4^. Follow-up studies of patients with nAMD found SF in over 35% after one year, 40–45% after 2 years, 56% after 5 years and up to 71% after 10 years^1,2,5–7^. It has been shown that the switch from a neovascular lesion to a mostly fibrotic lesion can happen within months despite optimal anti-VEGF treatment^8,9^. While anti-VEGF does not prevent SF development in all cases, formation of disciform scars may be delayed or aborted^6^. Hence, early diagnosis of eyes at risk of SF development is key to timely intervention and aggressive therapy. Ideally, diagnostic markers would allow risk stratification in treatment-naïve eyes, reliable predictors for SF development, however, are lacking. Moreover, the diagnosis of SF itself is challenging since interpretation of current gold standard imaging modalities such as color fundus photography (CFP) and fluorescein angiography (FA) is inherently subjective. Even on high-resolution spectral-domain optical coherence tomography (SD-OCT) diagnosing SF is not straight forward.

Novel imaging modalities such as OCT angiography (OCTA) or polarization-sensitive OCT (PS-OCT) allow a more in-depth analysis of macular neovascularization (MNV) and SF^10,11^. OCTA provides non-invasive and unobscured rendering of blood flow and identifies blood vessels down to the level of capillaries. Previous studies have used OCTA for qualitative evaluation of MNV lesions, but more recently detailed analysis using advanced evaluation software provided objective and quantitative assessment beyond the classification into patterns such as “dead tree” or seafan shape^12–18^. PS-OCT, an extension of conventional OCT technology, has the ability to automatically differentiate ocular structures based on tissue-specific contrast^11,19^. Identification of scar tissue by PS-OCT in the ophthalmic as well as the dermatologic setting has been demonstrated previously^20–23^. Detection of the polarization state of the back-reflected probing light beam allows differentiation of distinct physical properties in the human eye: birefringence, depolarization and polarization preservation. Subretinal fibrosis can be identified based on birefringence caused by interaction of collagen fibers with the probing light beam, while the retinal pigment epithelium (RPE) is inherently depolarizing owing to the polarization-scrambling effect of melanin granules within RPE cells^23–25^. Photoreceptors are polarization-preserving and do not alter the polarization state of the probing light beam.

Aim of this study was to use current goldstandard as well as novel imaging modalities in treatment-naïve eyes with nAMD to identify baseline predictors for SF.

## Materials and methods

### Study design and participants

This was a prospective longitudinal observational study conducted at the Department of Ophthalmology and Optometry and the Center for Medical Physics and Biomedical Engineering (CMPBE) at the Medical University of Vienna. The study protocol (clinicaltrials.gov identifier: NCT03838679) and procedures adhered to the tenets of the Declaration of Helsinki and were approved by the ethics committee of the Medical University of Vienna. Informed consent was obtained from each participant before inclusion.

Forty-five eyes of 45 participants with treatment-naïve nAMD were consecutively and prospectively enrolled. Patients were examined and treated according to a standardized treat-and-extend protocol using aflibercept^26^. Patients with previous anti-VEGF therapy, media opacification preventing high-quality imaging or retinal disease other than AMD were not included. Study visits were scheduled at baseline, months 1, 2, 3, 6 and 12, the total number of visits, however, was dependent on treatment response and there could be up to 13 visits within the 12-month study period.

All patients had a full ophthalmic examination including best-corrected visual acuity (BCVA) testing using the early treatment of diabetic retinopathy study (ETDRS) score, slitlamp and dilated fundus examination and SD-OCT imaging using the Spectralis HRA + OCT device (Heidelberg Engineering, Heidelberg, Germany) at every visit. OCTA imaging was performed at every visit using a novel swept-source optical coherence tomography angiography (OCTA) device (Plex Elite 9000; Carl Zeiss Meditec, Dublin, CA, USA) with an integrated eye-tracker with a 6 × 6 mm scan pattern centered on the fovea. Microperimetry (MP) was performed at baseline and months 3, 6 and 12 using the Micro Perimeter 3 (Nidek Tokyo, Japan).

Fluorescein angiography (FA) and indocyanine green angiography (ICGA) were performed at baseline and month 12 using the Spectralis HRA + OCT. CFP and PS-OCT imaging were performed at baseline and at months 1, 2, 3, 6 and 12. CFP imaging was performed using the integrated color fundus camera of the MP machine, which achieves high-quality true-color images. PS-OCT imaging was performed using a custom-built SD-OCT based investigational PS-OCT developed at the CMPBE incorporating a line-scanning laser ophthalmoscopy channel for real-time retinal tracking. The device operates at a center wavelength of 863 nm with a full-width half maximum bandwidth of 60 nm and acquires volume scans of 8 × 6 mm containing 250 B-scans with 1,024 A-scans in 4.5 s. Details of the device have been described previously^23,27^. Different information (reflectivity, phase retardation, optic axis orientation) is retrieved simultaneously from the backscattered probing light beam and is used for selective tissue detection.

### SF grading

The presence of SF was diagnosed based on CFP, FA and PS-OCT and was defined as follows: On CFP SF was apparent as well-defined, solid-appearing whitish or yellowish material^5,9,28^. In FA SF was characterized by early hypofluorescence and late staining^6,28^. In PS-OCT, SF was detected automatically based on tissue birefringence. For detection of birefringent tissue, we applied an algorithm that improves on our previous work^23^ by evaluation of the optic axis uniformity, a parameter that has higher values in fibrotic tissue and low values in surrounding non-birefringent tissue^22^. Details of the algorithm can be found in a recent publication^25^. MP was analyzed for cases with differential results in CFP, FA and PS-OCT. Since areas with SF have been shown to have significantly reduced retinal sensitivity, normal retinal sensitivity values were interpreted as unlikely to be SF in the respective area^29^.

### MNV lesion grading

Neovascularization was classified as type 1 (= sub-RPE), type 2 (= subretinal), mixed type (subretinal + sub-RPE), type 3 (= retinal angiomatous proliferation [RAP]) or polypoidal choroidal vasculopathy (PCV), based on FA, ICGA, OCTA and SD-OCT^30^.

SD-OCT images were evaluated for the presence of SRF, IRF and SHRM. SRF was defined as nonreflective space between the posterior boundary of the neuroretina and the RPE. IRF was defined as round, nonreflective to minimally reflective spaces within the neuroretina. SHRM was defined as hyperreflective material between the posterior boundary of the neuroretina and the RPE.

CFP was evaluated for the presence of retinal hemorrhage and the size was documented as either ≤ 1 disc area or > 1 disc area.

### Quantitative OCTA image analysis

Baseline OCTA volume scans of all patients were analyzed and divided into two groups: Eyes that developed SF within 12 months (SF group) and eyes without SF after 12 months (non-SF group).

Only well-centered volume scans with minimum signal strength of 7 (out of 10) and without motion artifacts were included in the study. Eyes without a clearly detectable MNV were excluded from the quantitative OCTA analysis.

The anterior and posterior border of the slab was adjusted to closely encompass the entire MNV complex using the built-in software of the Plex Elite 9000 device. The integrated projection artifact removal function was used to eliminate artifacts originating from retinal vessels anterior to the lesion.

**Figure 1:**
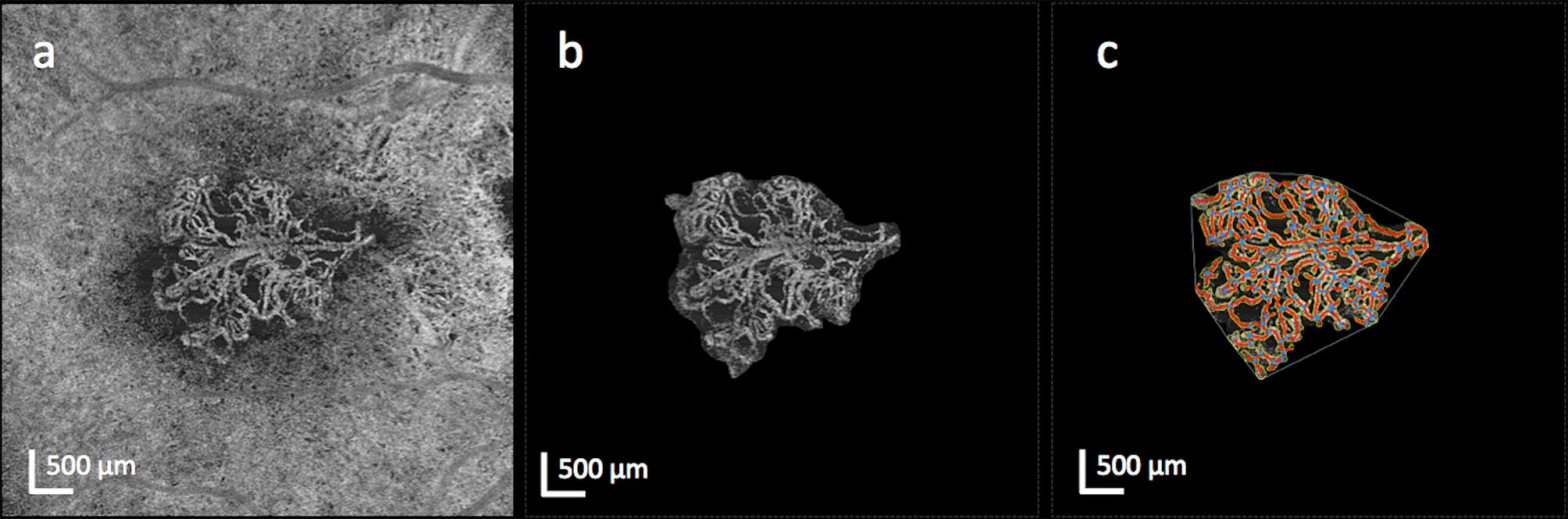
Representative swept-source optical coherence tomography angiography (OCTA) en face imaging of a left eye with a type 1 macular neovascularization (MNV) (**a**). After careful manual segmentation of the MNV lesion (**b**) in FIJI, the Angiotool program was used for quantitative analysis of the microvascular complex. The result of the analysis (**c**) shows the skeletonized vessels (red lines), junctions (blue dots), lesion area (outer white line) and endpoints (points of open-ended vessels).

For quantitative analysis of the vascular components of the MNV complex we used the publicly available image processing software FIJI (ImageJ 2.0.0, imagej.net) and the open-source and validated analysis software Angiotool (version 0.6a, https://ccrod.cancer.gov/confluence/display/ROB2/Downloads)^14–18^. After manual segmentation and background removal in FIJI, the segmented MNV lesions were quantitatively analyzed in Angiotool using the following parameters: The low threshold parameter was set between 9 and 35, depending on the brightness level of the OCTA en face image. The high threshold parameter was set to 255, vessel thickness was set between 3 and 6 depending on the vessel calibers within the MNV lesion and removal of small particles was set between 10 and 80 (Fig. 1). Angiotool identifies the vessel structure using a mostly automated analysis process with minimal user intervention required^16^. Identification and segmentation of the vessels is achieved by multiscale Hessian analysis and smoothing with a recursive Gaussian filter, followed by skeletonization and skeleton analysis^16^. A detailed explanation of the different Angiotool parameters can be found in previous publications^16,18^. In brief, the program gives the lesion area, the vessel area, equivalent to the area with flow signal within the MNV complex, the number of vessel junctions within the MNV complex, the total vessel length (sum of all vessels), the number of endpoints as well as mean lacunarity, reflecting the inhomogeneity of the neovascular structure. The junction density per unit vessel length was calculated as total number of junctions/total vessel length as described previously^31^. Additionally, vessel length density (total length of vessel/ vessel area) and endpoint density (total number of vessel endpoints/total length of vessel) were calculated as described previously^18^.

### Statistical analysis

Statistical analyses were performed using IBM SPSS statistics version 21 (IBM SPSS Statistics, IBM Corporation, Chicago, IL, USA). Descriptive statistics and Fisher’s exact test were used for qualitative findings. Independent samples Mann–Whitney U test was used to assess differences of quantitative parameters. No adjustment for multiple testing was performed, as the goals of the study are exploratory rather than confirmatory. A *p* value of < 0.05 was considered statistically significant.

## Results

We included 45 eyes of 45 patients [29 female (64%), 16 male (36%)] with treatment-naïve nAMD with a mean (± standard deviation [SD]) age of 77 (± 6) years. Mean (± SD) BCVA at baseline was a 68 (± 14) ETDRS letter score equivalent to LogMAR 0.35 (± 0.28). Twenty-six eyes (58%) were diagnosed with type 1 MNV, 5 eyes (11%) with type 2 MNV, 5 eyes (11%) with mixed type MNV, 8 eyes (18%) with type 3 MNV and 1 eye (2%) with PCV. Fifteen eyes (33%) were diagnosed with a retinal hemorrhage, 43 eyes (96%) had SRF, 27 eyes (60%) had IRF and 28 eyes (62%) had SHRM at baseline. After a 12-month follow-up period and a mean of 8.3 (± 1.6) intravitreal aflibercept injections, 8 eyes (18%) had developed SF. Baseline patient characteristics and differences between eyes with and without SF development are presented in Table 1.

**Table 1:** Patient characteristics and MNV types at baseline.

	No subretinal fibrosis (n = 37)	Subretinal fibrosis (n = 8)	*p* value
Age, years	78 (63–89)	78 (61–82)	0.760
Gender female/male, n (%)	22 (59%) / 15 (41%)	7 (88%) / 1 (13%)	0.226
Number of aflibercept injections	7 (7–12)	8 (7–11)	0.285
BCVA LogMAR	0.2 (0.1–1.0)	0.6 (0.1–1.1)	0.016*
BCVA (ETDRS letter score)	74 (36–84)	54 (30–74)	0.001*
Subretinal fluid present, n (%)	35 (95%)	8 (100%)	1.000
Intraretinal fluid present, n (%)	19 (51%)	8 (100%)	0.014*
SHRM, n (%)	20 (54%)	8 (100%)	0.017*
Retinal hemorrhage present, n (%)	11 (30%)	4 (50%)	0.410
**MNV type**			
Type 1, n (%)	26 (70%)	0	< 0.001*
Type 2, n (%)	3 (8%)	2 (25%)	0.211
Mixed type, n (%)	0	5 (63%)	< 0.001*
Type 3, n (%)	7 (19%)	1 (13%)	1.000
PCV, n (%)	1 (3%)	0	1.000
Subretinal MNV component present (type 2/mixed type), n (%)	3 (8%)	7 (88%)	< 0.001*

Independent samples Mann Whitney U test was used for continuous variables.

Fisher’s exact test was used for categorical variables.

The variables age, number of aflibercept injections, BCVA LogMAR and BCVA (ETDRS letter score) are given in median (minimum–maximum).

*BCVA* best corrected visual acuity, *LogMAR* Logarithm of the minimum angle of resolution, *ETDRS* early treatment of diabetic retinopathy research study, *SHRM* subretinal hyperreflective material, *MNV* macular neovascularization, *PCV* polypoidal choroidal vasculopathy.

There was no significant difference in age (*p* = 0.76), gender (*p* = 0.23) or number of injections (*p* = 0.29) between the SF and non-SF group or in the presence of SRF (*p* = 1.0) or retinal hemorrhage (*p* = 0.41) at baseline (Table 1). Two out of 15 eyes (13%) with retinal hemorrhage had an area of > 1 disc area, both of which were type 1 MNV and did not develop SF. Eyes in the SF group had worse baseline BCVA than eyes in the non-SF group [median (minimum–maximum) of 54 (30–74) versus 74 (36–84) ETDRS letter score; p = 0.001| and a higher prevalence of IRF (8 eyes (100%) versus 19 eyes (51%); *p* = 0.014) and SHRM at baseline (8 eyes (100%) versus 20 eyes (54%); *p* = 0.017). Type 1 MNV was the most common type in eyes without SF (26 eyes [70%]) and had a higher prevalence than in the SF-group (0 eyes [0%], *p* < 0.001). Mixed type MNV was the most common type in the SF-group (5 eyes [63%]) and had a higher prevalence than in the non-SF group (0 eyes [0%], *p* < 0.001). When combining all eyes with a subretinal MNV component (type 2 and mixed type MNV), there were more eyes in the SF-group (7 eyes; 88%) than in the non-SF group (3 eyes; [8%] *p* < 0.001).

**Figure 2:**
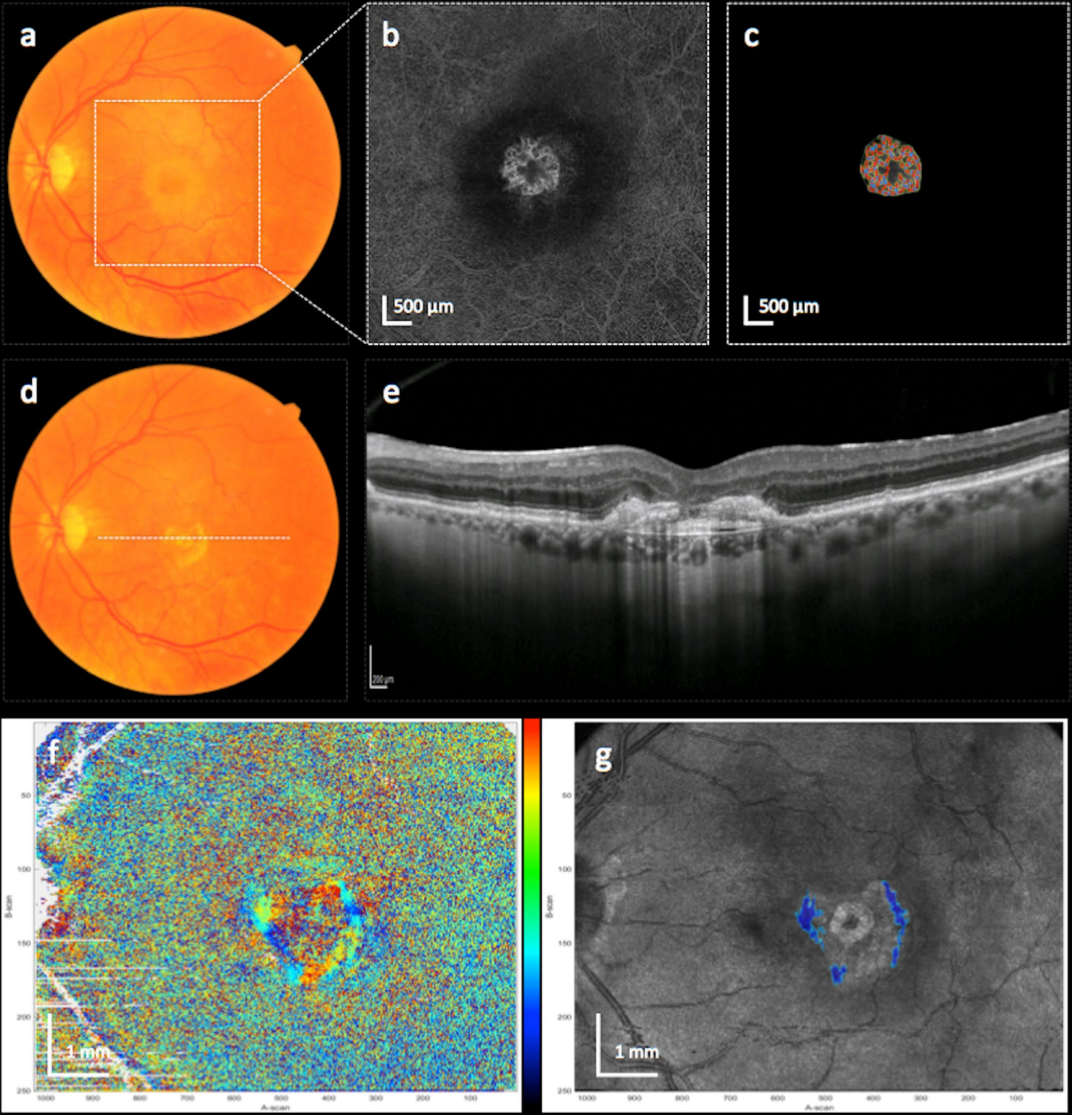
Left eye of a patient with a mixed type MNV, who developed subretinal fibrosis (SF). Figures show the MNV at baseline (**a**–**c**) and after 12 months (**d**–**g**). Color fundus photography at baseline (**a**) and after 12 months (**d**) shows the development of yellow subretinal material, colocalized with an area of subretinal hyperreflective material in the SD-optical coherence tomography (OCT) B-scan at month 12 (**e**). Axis orientation (**f**) and pseudo-SLO PS-OCT imaging (**g**) with automatically segmented fibrosis (highlighted in blue) illustrate the area of the subretinal fibrosis. Color bar indicates − 90 to + 90° for axis orientation. Baseline en face OCT angiography (**b**) shows a subfoveal circular neovascular net. The result of the quantitative vessel analysis using the Angiotool software is shown in (**c**). The location of the OCTA en face images is indicated by the white square in (**a**) and the location of the SD-OCT B-scan is indicated by the white dotted line in (**d**).

**Figure 3:**
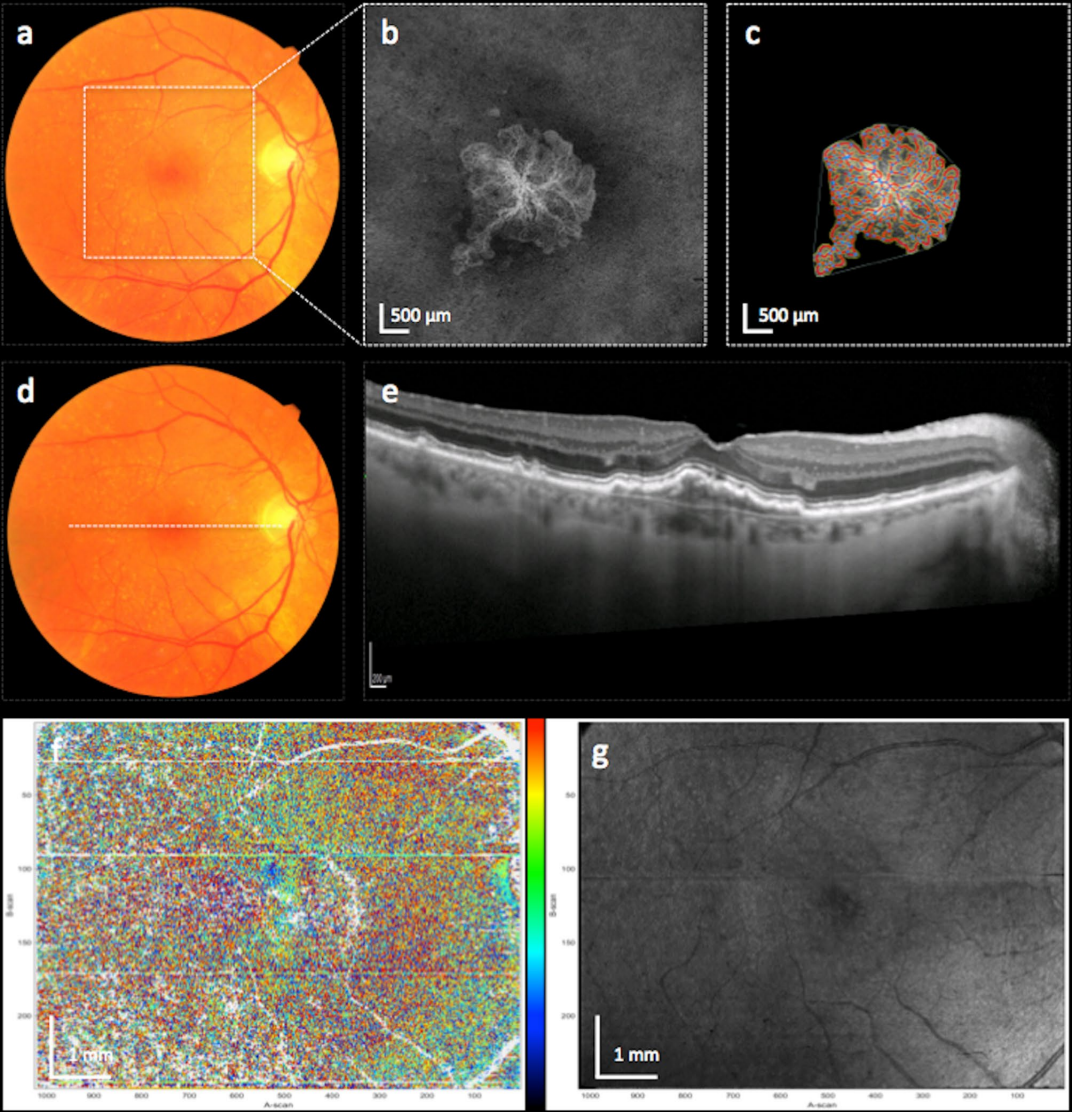
Right eye of a patient with a type 1 MNV, who did not develop subretinal fibrosis. Figures show the MNV lesion at baseline (**a**–**c**) and after 12 months (**d**–**g**). Color fundus photography at baseline (**a**) and after 12 months (**d**) show no presence of subretinal whitish-yellow material. SD-optical coherence tomography (OCT) at month 12 (**e**) shows a fibrovascular pigment epithelial detachment but no signs of exudation such as subretinal fluid or intraretinal fluid. Axis orientation (**f**) and pseudo-SLO PS-OCT imaging (**g**) show no presence of fibrosis. Color bar indicates − 90 to + 90° for axis orientation Baseline en face OCT angiography (**b**) as well as the result of the quantitative vessel analysis (**c**) using the Angiotool software is shown. The location of the OCTA en face images is indicated by the white square in (**a**) and the location of the SD-OCT B-scan is indicated by the white dotted line in (**d**).

Figures 2 and 3 show representative examples of eyes with and without SF development.

### Quantitative OCTA analysis

Thirty-three baseline OCTA volume scans of 33 eyes were included in the quantitative MNV analysis using Angiotool, 26 eyes from the non-SF group and 7 from the SF-group. In 12 out of the 45 eyes included in the study the MNV was not identifiable on OCTA at baseline. Eight of these eyes were diagnosed with a type 3 MNV (one of which developed SF), three eyes with a type 1 MNV and one eye with a PCV. Table 2 provides details about quantitative MNV parameters. We did not observe a significant difference in any of the evaluated parameters between eyes with and without SF development. A trend was observed towards a larger median total lesion area in the SF group than in the non-SF group (*p* = 0.120). Also, a trend towards a higher number of junctions (*p* = 0.120), a longer total vessel length (*p* = 0.090) and a higher number of endpoints (*p* = 0.090) was observed in the SF group, but none of the parameters reached statistical significance.

**Table 2:** Quantitative MNV parameters at baseline.

Quantitative MNV features, Median (minimum–maximum)	No subretinal fibrosis (n = 26)	Subretinal fibrosis (n = 7)	*p* value
Lesion area (mm^2^)	1.32 (0.16–14.43)	3.91 (0.75–9.46)	0.120
Vessels area (mm^2^)	0.59 (0.10–7.20)	1.76 (0.46–3.92)	0.143
Vessels percentage area (%)	51.6 (19.53–70.89)	45.04 (38.82–61.18)	0.120
Number of junctions (n)	56 (8–678)	166 (45–312)	0.120
Total vessel length (mm)	11.63 (2.27–138.76)	35.09 (9.45–71.18)	0.090
Vessel length density (mm/mm^2^)	19.28 (13.49–25.85)	19.79 (16.71–27.50)	0.813
Total number of endpoints (n)	21 (2–201)	56 (8–149)	0.090
Mean lacunarity	0.14 (0.08–0.68)	0.15 (0.06–0.29)	1.00
Junction density (n/mm)	4.82 (3.22–7.98)	4.65 (3.72–8.97)	0.330
Endpoint density (n/mm)	1.31 (0.60–3.14)	1.75 (0.85–2.09)	0.424

Independent samples Mann Whitney U test was used. *MNV* macular neovascularization.

### Fibrosis detection

**Figure 4:**
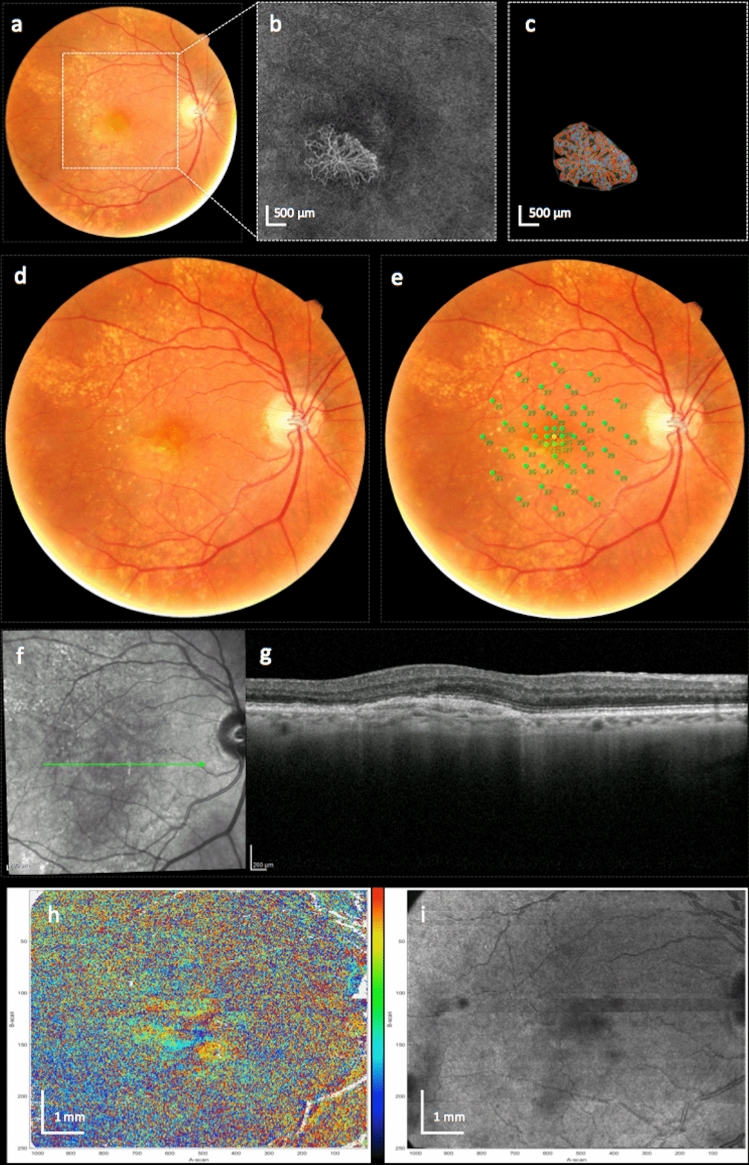
Right eye of a patient with a type 2 MNV and questionable development of subretinal fibrosis. Figures show the MNV lesion at baseline (**a**–**c**) and after 12 months (**d**–**i**). The patient had a small hemorrhage on color fundus photography at baseline (**a**) and developed questionable subretinal fibrosis (SF) after 12 months visible as faint yellowish subretinal material in (**d**). The area is colocalized with a fibrovascular pigment epithelial detachment in the SD-optical coherence tomography (OCT) B-scan at month 12 (**g**). The location of the B-scan is indicated by the green arrow in the infrared en face image (**f**). The baseline en face OCT angiography image (**b**) and the quantitative vessel analysis (**c**) using the Angiotool software is shown and the location of the OCT angiography en face images is indicated by the white dotted square in (**a**). Microperimetry (**e**) shows well-preserved retinal function indicated by green dots in the area with presumed fibrosis. Axis orientation (**h**) and pseudo-SLO PS-OCT imaging (**i**) do not confirm the presence of fibrosis. Using multimodal imaging the lesion was therefore graded as no fibrosis. Color bar indicates − 90 to + 90° for axis orientation.

Using CFP, FA and novel PS-OCT imaging, we identified 8 eyes with SF at month 12. Nine eyes were graded as fibrosis based on CFP alone and 8 eyes were graded as SF by FA and PS-OCT, respectively. In the one eye with differential results (SF in CFP, non-SF in FA and PS-OCT), retinal sensitivity in MP was preserved in the area of presumed SF, making the presence of SF highly unlikely (Fig. 4)^29^. After open adjudication, the expert graders (PKR, MS, MP and CKH) concluded that the lesion was a sub-RPE MNV, mimicking SF.

## Discussion

In this longitudinal observational study, we used novel SS-OCTA imaging and image analysis software to analyze in detail the microvascular features of treatment-naïve nAMD and identify baseline predictors for SF. CFP and FA were supplemented by PS-OCT for automated detection of SF. Interestingly, the quantitative analysis of MNV lesions did not reveal baseline predictors for SF development.

Querques et al. investigated eyes with nAMD with a history of anti-VEGF therapy and without neovascular activity for at least 6 months without treatment. The authors found that eyes with SF, termed “fibrocellular phenotype”, had a significantly lower perfusion density than eyes without SF, termed “fibrovascular phenotype”^32^. Similarly, Miere et al. used OCTA to investigate eyes with SF and classify the patterns of the observed vascular structures^33^. Despite the presence of preserved MNV vasculature within SF, the vascular density was reduced in their study^33^. In contrast to these studies, we investigated eyes in a treatment-naïve condition and did not find a microvascular difference between eyes with and without SF development after 1 year, suggesting that the differences in microvascular features between these groups develop over time and may not be present at first presentation. In fact, previous studies have shown vascular flow remodeling induced by recurrent anti-VEGF therapy as well as distinct growth patterns of MNV lesions with treatment^12,13^. Xu et al. observed growth of MNV lesions in 80% of cases in a mixed group of previously treated and treatment-naïve type 1 MNV despite ongoing anti-VEGF treatment, illustrating continuous change over time^12^. Miere et al. observed a change in the pattern of the MNV lesion with anti-VEGF therapy in 8 out of 9 eyes (89%) in a treatment-naïve group versus 3 out of 8 eyes (38%) in a previously treated group of patients with nAMD^13^. These studies suggest an impact of anti-VEGF treatment on the morphology of MNV lesions, which is why we chose to only include treatment-naïve eyes in our study to exclude this confounding factor. We observed a trend towards larger lesion area, a higher number of junctions, a longer total vessel length and a higher number of endpoints at baseline in the SF group, but none of these parameters reached statistical significance (Table 2). These non-significant outcomes may have been driven by the large spread of distribution in the non-SF group.

When investigating retinal structural findings at baseline we found that the presence of IRF (*p* = 0.014) was more common in the SF group. While SRF was present in almost all eyes in both groups at baseline, IRF was observed only in 51% of cases in the non-SF group but in all eyes with SF development (Table 1). Intraretinal fluid may be found more often in eyes with severe damage to the outer retina, allowing fluid from the subretinal space to enter the retinal tissue and accumulate as intraretinal cysts. This may also explain why BCVA in these eyes was worse at baseline than in eyes without SF development.

SHRM was observed more often in the SF than in the non-SF group (*p* = 0.017), in line with previous studies such as CATT^34^. SHRM in the non-SF group at baseline may be composed solely of exudate and AMD-associated debris, while in the SF group SHRM may be composed primarily of a fibrovascular complex, transforming into a fibrotic lesion under anti-VEGF therapy^8,34,35^.

There was no difference in distribution of eyes with SRF between the SF and the non-SF group in our study (*p* = 1.000), while in the CATT study the presence of SRF has been identified as a risk factor for SF^5^. One reason may be that in CATT, time-domain OCT was performed at baseline and minute amounts of SRF may have been missed in some eyes.

Counterintuitively, there was no difference in distribution of eyes with retinal hemorrhage at baseline between the SF and the non-SF group in our study (*p* = 0.410). In CATT, retinal hemorrhage larger than one disc area was a significant risk factor for scar development after 5 years^5^. Interestingly, only two eyes in our study had a retinal hemorrhage > 1 disc area, both of which had a type 1 MNV and did not develop SF. Another reason may be the slightly higher number of eyes with type 3 MNV in our study (n = 8; 18%) compared to CATT (n = 126 of 1183; 10.7%), which are more likely to be associated with a hemorrhage at baseline and less likely to develop scarring^36^. The relatively small total number of eyes with SF in our study must be mentioned as another possible cause for this finding.

Confirming previous observations, we found that a subretinal lesion component (type 2 or mixed type MNV) is more common in eyes with SF development, which may suggest that not the microvascular structure but the location of the MNV complex (sub-RPE versus subretinal) may be more critical for SF development^5,6,9^. This may be explained by a protective effect of the overlying RPE in type 1 lesions. In 2 of the type 2 MNV cases without SF development we observed RPE migration towards the MNV and conversion of the type 2 to a type 1 MNV, as described previously by Dolz-Marco et al.^37^. In their study they demonstrated, how early type 2 MNV lesions under anti-VEGF treatment can be enveloped by RPE cells and transformed into type 1 MNV, resulting in reduced outer retinal damage and preserved retinal function. In eyes with SF secondary to type 2 or mixed type MNV we did not observe a continuous RPE layer in the area of SF. We hypothesize that instead of forming a barrier between the MNV lesion and the neuroretina, the RPE may have undergone “epithelial-mesenchymal transition” in these eyes^35^. In this reaction, triggered by signal molecules such as transforming growth factor beta, the RPE undergoes a transition from an epithelial cell type with a typical cell polarity to a mesenchymal cell type, acquiring features such as extracellular matrix deposition and SF development^35^.

Limitations of this study include a small number of eyes with SF, a large spread of distribution for some of the quantitative parameters and the limited follow-up of 12 months. Additionally, the Angiotool program also has limitations in accuracy of vessel detection in the MNV complex. Positive aspects include the use of a high-quality SS-OCTA device operating at longer wavelength, allowing deeper penetration in tissue and better visualization of the MNV complex. Furthermore, we used PS-OCT and MP as ancillary imaging methods to reliably detect SF, which proved particularly helpful in challenging cases.

In conclusion, using novel multimodal imaging and analysis software, we did not identify quantitative microvascular indicators for SF development in treatment-naïve eyes with nAMD. The presence of a subretinal MNV component, IRF and SHRM on SD-OCT and low baseline BCVA, however, are easily identifiable parameters indicating an increased risk and warranting close follow-up and intensified anti-VEGF therapy.
